# Potential Role of Thymosin Beta 4 in Liver Fibrosis

**DOI:** 10.3390/ijms160510624

**Published:** 2015-05-08

**Authors:** Jieun Kim, Youngmi Jung

**Affiliations:** 1 Department of Integrated Biological Sciences, Pusan National University, 63-2 Pusandaehak-ro, Kumjeong-gu, Pusan 609-735, Korea; E-Mail: jieun@pusan.ac.kr; 2 Department of Biological Sciences, Pusan National University, 63-2 Pusandaehak-ro, Kumjeong-gu, Pusan 609-735, Korea

**Keywords:** thymosin beta 4, liver fibrosis, hepatic stellate cells

## Abstract

Liver fibrosis, the main characteristic of chronic liver diseases, is strongly associated with the activation of hepatic stellate cells (HSCs), which are responsible for extracellular matrix production. As such, investigating the effective regulators controlling HSC activation provides important clues for developing therapeutics to inhibit liver fibrosis. Thymosin beta 4 (Tβ4), a major actin-sequestering protein, is known to be involved in various cellular responses. A growing body of evidence suggests that Tβ4 has a potential role in the pathogenesis of liver fibrosis and that it is especially associated with the activation of HSCs. However, it remains unclear whether Tβ4 promotes or suppresses the activation of HSCs. Herein, we review the potential role of Tβ4 in liver fibrosis by describing the effects of exogenous and endogenous Tβ4, and we discuss the possible signaling pathway regulated by Tβ4. Exogenous Tβ4 reduces liver fibrosis by inhibiting the proliferation and migration of HSCs. Tβ4 is expressed endogenously in the activated HSCs, but this endogenous Tβ4 displays opposite effects in HSC activation, either as an activator or an inhibitor. Although the role of Tβ4 has not been established, it is apparent that Tβ4 influences HSC activation, suggesting that Tβ4 is a potential therapeutic target for treating liver diseases.

## 1. Introduction

Liver disease, including liver cancer, is a major cause of morbidity and mortality worldwide [1,2,3,4]. The main cause of liver disease is viral, autoimmune, drug/toxin, alcohol and nonalcoholic fatty liver disease [5]. Liver fibrosis is the main hallmark of most chronic liver diseases and can progress into end-stage cirrhosis showing the altered hepatic function and the dysregulated regenerative process with formation of fibrous bands [6]. Liver fibrosis typically occurs in response to hepatic injury; however, progressive and/or severe damage leads to the massive deposition of extracellular matrix (ECM) proteins, promoting a distorted architecture of the liver [7]. These fibrous matrix proteins are mainly produced by activated hepatic stellate cells (HSCs), which are transdifferentiated from quiescent HSCs. The activated HSCs, which look like a myofibroblast, with long processes without lipid droplets, contribute to liver fibrosis by accumulating ECMs [8]. However, the underlying mechanisms of HSC activation are poorly understood, and no effective treatments have been found.

Thymosin beta 4 (Tβ4) is a highly conserved G-actin sequestering protein that exerts a broad range of functions, such as the promotion of cell migration, angiogenesis, and ECM synthesis [9]. Recently, aberrant Tβ4 has been shown to play a key role in the development of organ fibrosis [10]. In addition, it has been reported that Tβ4 is expressed in activated HSCs during liver fibrosis and that it influences the activation of HSCs [11,12]. These findings suggest that Tβ4 is involved in the pathogenesis of liver fibrosis. Therefore, we review the functions and the related pathway of Tβ4 in several types of tissues and/or cells and describe the endogenous and exogenous actions of Tβ4 in liver to suggest how Tβ4 is potentially involved in the pathogenesis of liver fibrosis.

## 2. Thymosin Beta 4

Thymosin isolated from the thymus gland was first described in 1966 by Goldstein and White [13]. The β-thymosin family is composed of biochemically and functionally distinct polypeptides. Tβ4, Tβ10 and Tβ15, among the known family members, are found in humans [9]. The most abundant peptide in this family, Tβ4, is a highly conserved and water-soluble, acidic polypeptide consisting of 43 amino acids and a molecular weight of 4964 Da [14]. Tβ4 is selectively crosslinked by factor XIIIa to various molecules, including actin, collagen, and fibrin [9]. It also acts as a major actin-sequestering molecule in all eukaryotic cells and is a potent regulator of actin polymerization in mammals [15]. Emerging evidence suggests that Tβ4 is involved in a number of cellular responses, such as angiogenesis, wound healing, hair growth, apoptosis, and inflammation [16,17,18,19]. Tβ4, released by platelets in the region of injured dermis, can accelerate dermal wound healing by promoting cell migration, accelerating collagen deposition, and inhibiting both inflammation and apoptosis [20].

Tβ4 promotes the survival of cardiac myocytes after ischemia, an effect that is mediated by the increased expression of vascular endothelial growth factor (VEGF) and activation of integrin-linked kinase (ILK) [21]. Recent studies have shown that Tβ4 is overexpressed in malignant tumors, and it has been suggested that it is associated with metastatic capability and angiogenesis [16,22,23,24,25]. Induced expression of Tβ4 has been shown to enhance tumor growth and metastasis in melanoma cell lines and mouse fibrosarcoma [26]. In addition, Tβ4-overexpressing human colon cancer cells have exhibited increased growth and invasion in transplanted mice with those cancer cells [25]. Huang *et al.* [27] revealed that Tβ4 overexpression promoted cell invasion and migration through the ILK/AKT/β-catenin signaling pathway and triggered epithelial-to-mesenchymal transition (EMT) in colorectal carcinoma. Tβ4 also acts as a hypoxia-responsive regulator that controls cancer cell migration in angiogenesis and tumor metastasis [28].

These various cellular responses are regulated by the Tβ4-mediated expression of several genes, such as specific proteases, laminin-5, and several inflammatory cytokines and chemokines. Tβ4 increased the expression of laminin-5 and transforming growth factor-β (TGF-β) in cultured corneal cells, which in turn upregulated laminin-5- and TGF-β-induced cell migration and collagen synthesis, respectively [29]. Tβ4 has been shown to influence the Wnt signaling pathways by regulating the activation of glycogen synthase kinase-3 (GSK-3) in the migration of gastric cancer cells [30]. Hepatocyte growth factor (HGF) promotes the upregulation of Tβ4, which influences wound healing in human umbilical vein endothelial cells [31]. Thus, because Tβ4 exerts a broad spectrum of functions by interacting with various molecules, it is important to research how Tβ4 is regulated and what its targets are, in order to understand the action mechanism of Tβ4.

## 3. Liver Fibrosis and Hepatic Stellate Cells

Liver fibrosis is a common wound-healing response to liver injury [32]. However, persistent and/or severe damage, such as continuous alcohol consumption, viral infection, toxins, and high-fat diets, impair the repair response, leading to the development of fibrotic scar and chronic wounds characterized by excessive inflammation and collagen deposition and the dysregulated proliferation of several types of hepatic cells [33]. The major type of cells contributing to fibrosis is HSC [7]. In the liver, HSCs reside in the space of Disse between hepatocytes and sinusoidal endothelial cells, and they extend their dendritic processes along the walls of the sinusoids. HSCs undergo transdifferentiation from “quiescent” HSCs into “activated/myofibroblastic” HSCs during hepatic injury [32]. In the quiescent stage, HSCs store perinuclear retinoid (vitamin A) droplets and synthesize glial fibrillary acidic protein (GFAP) [34]. As they are activated, retinoid droplets and GFAP are gradually lost, followed by development into myofibroblast-like cells with increased synthesis of ECM proteins and α-smooth muscle actin (α-SMA) [33]. These activated HSCs migrate and proliferate effectively in response to a variety of cytokines and growth factors stimulated by liver injury [35]. The transdifferentiation, proliferation, and ECM production of HSCs are the important events that occur during liver fibrosis. Therefore, it is believed that developing effective strategies to suppress these events will reduce fibrosis or prevent a progression into chronic liver injury. In particular, many studies have been conducted on the transdifferentiation of quiescent HSCs into activated HSCs, as this process has been considered to be a possible therapeutic target to inhibit liver fibrosis [36,37].

Several factors, such as TGF-β, platelet-derived growth factor (PDGF), phosphatidylinositol 3-kinase (PI3K)/protein kinase B (AKT), and hedgehog (Hh), which have been reported to regulate hepatic fibrogenesis, are highly expressed in the damaged liver and stimulate the transition of quiescent HSCs and/or promote the proliferation of newly generated HSCs [35,38,39,40]. TGF-β, a well-known fibrosis-stimulating factor, plays a critical role in regulating HSC activation and ECM production in HSCs [41]. The PI3K/AKT pathway is strongly activated by the well-known mitogen PDGF and is required for the survival and proliferation of HSCs [38,39,42]. Hh signaling regulates the proliferation and activation of HSCs, leading to liver fibrosis [43,44]. In addition, TGF-β and Hh have been known to promote epithelial-to-mesenchymal transition (EMT), which is an important pathogenic pathway that contributes to the excessive accumulation of activated HSCs, leading to collagen deposition during liver fibrosis [44,45].

## 4. Thymosin Beta 4 and Liver Fibrosis

Although Tβ4 is known to be expressed in the liver, the types of hepatic cells that specifically express Tβ4 have not been well established. Based on the immunochemical data, Nemolato *et al.* [46] reported that hepatocytes expressed Tβ4 in the healthy human liver, whereas Paulussen *et al.* [47] reported that Kupffer cells (hepatic macrophages) express Tβ4 in the damaged liver. In addition, increased expression of Tβ4 has been reported in human hepatocellular carcinoma [48] and hepatoblastoma [49]. Recent studies with more evidence have revealed that activated HSCs express Tβ4 in chronically damaged livers [11,12]. Because the activated HSCs are the main source of collagen production, investigation into the potential role of Tβ4 in the activation of HSCs in the liver is needed. However, conflicting results in the regulation of Tβ4 for HSC activation, inhibition, or promotion have been reported. Therefore, it is necessary to first examine the types of cells that express Tβ4 in order to identify the exogenous and endogenous roles of Tβ4 in the liver.

### 4.1. Role of Exogenous Tβ4

Several studies have suggested that Tβ4 treatment has an antifibrotic effect in the liver. Barnaeva *et* *al.* [50] demonstrated that Tβ4-treated HSCs showed an upregulation of HGF and a downregulation of the PDGF-β receptor at the RNA levels. The researchers have suggested that exogenous Tβ4 has an antifibrogenic function in human HSCs because the level of HGF expression is reduced in fully activated HSCs [51] and PDGF-β receptor is required for the activation of HSCs [52]. However, α-SMA, a well-known marker of activated HSCs [53], and β-catenin and GSK-3β, members of the Wnt pathway involved in the transdifferentiation of HSCs [54,55], were upregulated in a Tβ4-dose-dependent manner. In addition, the expression of other ECM components, such as collagen1α2, tissue inhibitor of metalloproteinase-1, and matrix metalloproteinase (MMP)-2, was not changed by Tβ4. Although these results suggest that downregulation of the PDGF-β receptor by Tβ4 prevents the proliferation of HSC-derived myofibroblasts, there has been no explanation for the increased expression of other ECM members, especially α-SMA, by Tβ4. Further studies are required to prove the disparity in the actions of Tβ4 in PDGF-β receptor and α-SMA, as both have been considered hallmarks of HSC differentiation into myofibroblasts. In 2011, Reyes-Gordillo *et al.* [56] reported that Tβ4 treatment prevented PDGF-ββ-dependent proliferation and migration of cultured human HSCs by inhibiting PDGF-ββ-dependent phosphorylation of AKT. In these studies, the researchers demonstrated that Tβ4 interrupted the movement of AKT into PI3K, blocking the phosphorylation of AKT by PI3K in HSCs treated with PDGF-ββ [56]. While exogenous Tβ4 treatment successfully reduced the proliferation and migration of HSCs stimulated by PDGF-ββ, when the activated HSCs were treated with Tβ4, these cells also showed decreased or unchanged expression of the genes under examination. Hence, it is possible that endogenous Tβ4 is combined with exogenous Tβ4 treatment, and that too high of a concentration of this combined Tβ4 desensitizes the Tβ4 signaling pathway, eventually blocking the proliferation of HSCs. In these two studies, it is noted that Barnaeva and Reyes-Gordillo did not examine the endogenous expression and role of Tβ4 in HSCs. Nevertheless, Reyes-Gordillo *et al.* [57] demonstrated in 2012 that exogenous Tβ4 treatment ameliorated acute liver damage induced by a single injection of carbon tetrachloride (CCl_4_) in rats. The liver morphology is improved in the CCl_4_ + Tβ4-treated group compared to the CCl_4_-treated group, assessed by hematoxylin and eosin staining. The hepatic protective effect of exogenous Tβ4 is mediated by inhibiting the upregulation of fibrotic markers (PDGF-β receptor, α-SMA, collagen1α2 and fibronectin) and methyl-CpG-binding protein 2 (MeCP2) and the downregulation of adipogenic transcription factor peroxisome proliferator-activated receptor (PPARγ) at the RNA level. PPARγ expressed by quiescent HSCs is reduced by MeCP2 in activated HSCs [58]. The researchers suggested that Tβ4 maintained the expression level of PPARγ by reducing MeCP2 expression, thus contributing to maintaining the quiescent phenotypic state of HSCs in rat livers [57]. However, the repair response, including activation of HSCs and inflammation, is a necessary process in the damaged liver [7]. The liver’s health is dictated by a balance between the factors that regulate injury and repair. Liver injury stimulates a repair response, and when the repair response is inadequate, tissue damage persists and can progress. Therefore, defective repair responses permit mild liver injury to progress to cirrhosis [33]. Liver damage caused by a single CCl_4_ injection stimulated a repair response, and Tβ4 treatment clearly showed the improved morphology of a liver treated with CCl_4_, indicating that Tβ4 promotes liver regeneration. Therefore, the mechanism by which the repair process occurs without the activation of HSCs should be identified.

Taken together, exogenous Tβ4 has a role in attenuating liver fibrosis, a process that seems to be mediated by preventing the proliferation and migration of HSCs [50,56,57]. However, it is first necessary to investigate the types of cells that express endogenous Tβ4 in the liver, as well as whether endogenous Tβ4 is actually expressed in activated or inactivated HSCs, in order to understand the role and mechanism underlying the effects of Tβ4 in the liver.

### 4.2. Role of Endogenous Tβ4

As the expression and function of Tβ4 have been investigated recently in the liver, research on the types of cells that express Tβ4 has attracted attention. Nemolato *et al.* [46] and Paulussen *et al.* [47] reported the expression of Tβ4 in hepatocytes and Kupffer cells (CD 11b- or CD 68-positive cells), respectively. Tβ4 expression these cells was assessed by single immunostaining for Tβ4. Tβ4 was suggested to indirectly control the migration of Kupffer cells, based on its ability to regulate the actin polymerization. [47]. Henkel *et al.* [59] also showed that Kupffer cells express Tβ4 in liver sections of patients with hepatitis C virus (HCV), as examined by immunostaining. They suggested Tβ4 as a new potential biomarker for inflammatory hepatic lesions, although Tβ4 at the RNA level was not related with the degree of fibrosis [59]. However, they presented the Tβ4-positive cells in HCV-infected liver only, although they examined the RNA level of Tβ4 in several kinds of liver diseases. In addition, the effect of Tβ4 expression in Kupffer cells was not investigated. Recently, Xiao *et al.* [11] and Kim *et al.* [12] provide direct evidence that activated HSCs express Tβ4 *in vivo* and *in vitro*. However, Xiao and Kim suggested directly opposite interpretations of the role of Tβ4 in the liver. Xiao *et al.* [11] demonstrated that LX-2 cells, the activated human HSC line, strongly expressed Tβ4, and that depletion of Tβ4 promoted the proliferation and migration of LX-2 cells via the activation of the PI3K/AKT signaling pathway. AKT (Ser473 and Thr308) and PI3K signaling components (p-85 regulatory subunit, PDK1 and GSK-3β) were phosphorylated in the Tβ4-suppressed LX-2 cells by small interfering RNA (siRNA) [11]. They also showed that Tβ4 was downregulated in both livers of bile-duct-ligated (BDL) rats and patient serum with cirrhosis. Bile duct ligation is a well-established model, in which the accumulated myofibroblasts or the activated HSCs produce excessive ECMs, leading to fibrosis [60]. Therefore, the livers of those rats should have displayed increased Tβ4 expression, because the activated HSCs proliferate abundantly in those livers [61,62,63] and Xiao *et al.* reported an upregulation of Tβ4 in these cells [11]. However, Tβ4 levels decreased in the BDL livers, although it was difficult to compare Tβ4 expression in a BDL liver with that of a healthy liver without evidence of Tβ4 expression in the livers of control rats. The researchers also suggested that Tβ4 depletion promoted HSC activation by exhibiting increased protein levels of vimentin and α-SMA protein. If Tβ4 inhibited HSC activation, higher levels of Tβ4 should have been expressed in the inactivated HSCs, and HSCs should have been activated in the absence or lower expression of Tβ4. However, there was no explanation for how the activated HSCs expressed Tβ4.

Kim *et al.* [12] recently suggested that activated HSCs express Tβ4, which regulates the activation of HSCs. In their studies, Tβ4 expression was upregulated in the fully activated LX-2 cells, the activated primary mouse HSC, the fibrotic livers of human patients, and the CCl_4_-induced fibrotic livers of mice. The suppression of Tβ4 by siRNA also induces the downregulation of myofibroblastic markers, such as TGF-β, α-SMA, collagen1α1, and vimentin, and the upregulation of the inactivation markers of HSCs, PPARγ and GFAP, with increased numbers of cytoplasmic lipid droplets in LX-2 cells and primary human HSCs [12]. Thus, these results suggest that activated HSCs express Tβ4 in chronically damaged livers, and that this endogenous expression of Tβ4 regulates HSC activation. To support the data for a higher level of Tβ4 expression in the chronically damaged liver, the researchers examined prolyl oligopeptidase (POP) activity in the livers of mice. POP degrades Tβ4 into *N*-acetyl-seryl-aspartyl-lysyl-proline (Ac-SDKP), which exhibits antifibrotic actions. Ac-SDKP is further degraded into an inactive metabolite by angiotensin-converting enzyme [64]. Kim *et al.* showed that POP activity decreased in CCl_4_-treated mice, suggesting that this decrease in POP contributed to the accumulation of Tβ4 in the damaged liver. Chen *et al.* [65] also supported the notion that preservation of basal Ac-SDKP attenuates CCl_4_-induced fibrosis in the rat liver; in their research, Tβ4 RNA level was unchanged and POP activity was alleviated, in the livers of rats treated with CCl_4_ for six weeks. The reduction in POP activity resulted in lower production of Ac-SDKP and higher expression of Tβ4 in livers with chronic injury. In addition, the treatment of Tβ4 with a POP inhibitor significantly increased renal fibrosis in the mice [10]. However, Kim *et al.*’s experimental system did not investigate the effect of exogenous Tβ4 or the mechanism regarding how Tβ4 regulates the activation of HSCs. Thus, there is no clear explanation for the disparity in the results of Xiao’s and Kim’s research. Further studies using transgenic mice overexpressing Tβ4 might be helpful for obtaining answers to those questions.

Although the effect of Tβ4 in liver fibrosis remains unclear, it is clear that Tβ4 is the potential key regulator of HSC activation, and that it can play an essential role in liver disease progression or prevention. Therefore, further studies are required to demonstrate the functions of endogenous Tβ4 in the liver for its future therapeutic application.

### 4.3. Possible Signaling Pathways of Tβ4 in Liver Fibrosis

The PI3K/AKT signaling pathway has been suggested as one of possible targets of Tβ4 in regulating the proliferation and migration of HSCs [11]. In this research, the suppression of Tβ4 induces HSC proliferation and migration by activating the PI3K/AKT pathway. In the cardiac repair process, Tβ4 promotes the migration and survival of cardiac cells by activating ILK [21]. Tβ4 also triggers an EMT in colorectal carcinoma by upregulating ILK [27]. The activity of ILK in these processes is regulated in a PI3K-dependent manner [66,67]. Activated PI3K stimulates ILK activity, which stimulates the phosphorylation of two downstream targets, AKT and GSK-3β. Although the effects brought by the Tβ4-PI3K/AKT pathway are varied depending on the cell types, it seems to be associated with cell response to Tβ4 stimulation.

In renal fibrosis, Tβ4 is upregulated in glomerulosclerosis and is required for the angiotensin-II-induced expression of PAI-1 (plasminogen activator inhibitor-1) [10]. PAI-1, a major inhibitor of both tissue-type plasminogen activator and urokinase-type plasminogen activator, is a key regulator of fibrinolysis by plasmin. Plasmin directly or indirectly degrades ECM components through the activation of MMPs [68]. Thus, PAI-1 influences organ fibrogenesis by impairing the plasminogen activator. Recent studies have shown that liver damage and fibrosis are ameliorated in PAI-1-deficient mice with BDL [69]. However, in PAI-1-knockout mice after chronic CCl_4_ exposure, these injuries are dramatically enhanced [70]. Although these studies reported different results depending on the experimental animal model used, the relationship between Tβ4 and PAI-1 might provide clues for understanding the mechanism of Tβ4 in the liver.

## 5. Conclusions

Liver fibrosis is the main characteristic of most chronic liver diseases, and HSCs are known to be the major source of fibrous matrix production. Therefore, the study of the effective regulator that influences the activation of HSCs is considered to provide important clues for developing therapeutics to inhibit liver fibrosis. As discussed above, increased levels of Tβ4 are associated with chronic liver disease and are involved in liver fibrosis by regulating the proliferation and activation of HSCs. Although it has not been established whether Tβ4 promotes or inhibits the activation of HSCs, it is obvious that Tβ4 is involved in the pathogenesis of liver fibrosis. The involvement of Tβ4 in fibrogenesis has been reported in several organs and its effect seems to be dependent on the cellular and patho-physiological conditions, implying that the endogenous expression of Tβ4 may be related to disease progression or prevention. The exogenous Tβ4 peptide is reported to improve cardiac function by rescuing the myocardium in patients with acute myocardial infarction [71], promoting the wound healing process in venous ulcer patients [72], and exerts anti-fibrotic effect in mice. However, the investigation of the therapeutic potential of Tβ4 should be preceded by an understanding of the role of endogenous Tβ4 and its interactions with other partners to develop safe and effective treatment for liver disease. Therefore, understanding the potential effects and regulatory mechanism of Tβ4 in liver fibrosis might help to provide a novel treatment for patients with liver fibrosis.
